# The impact of peripheral arterial disease on exercise tolerance and quality of life in the elderly and the role of cardiovascular physiotherapy: review article

**DOI:** 10.1590/1677-5449.200117

**Published:** 2021-04-28

**Authors:** Ana Leticia Gonçalves Lourenço, Josicléia Leôncio da Silva, Jéssica Costa Leite

**Affiliations:** 1 Centro Universitário UNIFACISA, Departamento de Fisioterapia, Campina Grande, PB, Brasil.

**Keywords:** peripheral arterial disease, exercise tolerance, quality of life

## Abstract

The primary symptom of peripheral arterial its intermittent claudication; a condition that causes functional disabilities, compromising quality of life. This review aimed to survey the impacts of this disease on the elderly, investigating possible contributions that cardiovascular physiotherapy has to offer. Searches were run on the MEDLINE, LILACS, SciELO, Scopus, Science Direct, and PEDro databases, identifying 7,587 studies. Seven of these met the eligibility criteria and were grouped and analyzed according evidence level, recommendation grade, and methodological quality. It was observed that this disease is responsible for considerable impact on exercise tolerance and quality of life. Regarding the therapeutic approach to these outcomes, the studies reported that there were improvements in walking and quality of life, increased functional capacity, and reduced pain. With regard to the treatment modality, most research included aerobic exercises.

## INTRODUCTION

Peripheral arterial occlusive disease can cause partial or total obstruction of the arteries of the lower limbs, provoking reduced blood flow to the extremities. In the most critical cases there is a risk of limb amputation if revascularization is not possible.^1^^,^ 2

Approximately 20% of the population affected by this disease are over the age of 65. In Brazil, it is estimated that 0.053% of the population is diagnosed with peripheral arterial disease (PAD) annually, including men aged from 55 to 74 years and women from 65 to 74 years. The disease can course asymptomatically in up to 80% of cases, delaying early diagnosis and making it more difficult to make a diagnosis, compromising prognosis as a result.^3^^,^ 4

Major risk factors for PAD include diabetes, smoking, hypertension, and dyslipidemia, which are all conditions that predominate in the elderly population.^5^ The primary element in clinical presentation is intermittent claudication, affecting around one third of patients. Other frequent symptoms include cramps, pain, and tiredness in the lower limbs, which worsen when walking and are relieved at rest.^6^

At more advanced stages of PAD, tissue necrosis can occur, greatly increasing the risk of losing the limb involved.^6^ All of these factors mean that PAD is responsible for impairment of patients’ functional capacity and reduced quality of life (QoL).^1^^,^ 3

It is therefore recommended that these people undergo early vascular assessment, aiming to determine their functional capacity and exercise tolerance. This can be achieved using direct measures, such as a treadmill test or the 6-minute walk test (6WT), or using indirect measures, employing specific scales.^7^ QoL can be assessed using questionnaires that cover physical, psychological, and social aspects and the subject’s self-perception.^8^

In this context, physiotherapy has an important role to play in PAD treatment, because it can be used during the preoperative period, with the objective of controlling pain, reducing swelling, increasing amplitude of movements, stimulating mobility, and providing health education, and also during the postoperative period, with the objective of promoting increased muscle strength, amplitude of movement, and functional capacity, using resistance exercises, aerobics, and flexibility training.^2^

In view of the above, and considering the increasing numbers of elderly people with PAD in Brazil, research into the subject seeking information of existing therapies and on the changes provoked by the disease can help to increase treatment success. This study was therefore conducted to review the literature, attempting to determine the impact of PAD on exercise tolerance and QoL in the elderly, in addition to investigating the possible contributions that physiotherapy can make towards minimizing these changes.

## METHODS

This is an integrative literature review based on a search for articles indexed on the MEDLINE/Pubmed, LILACS, SciELO, Scopus (Elsevier), Science Direct, and PEDro databases. Manual searches were also performed of the references of the articles included in this study and in previously published integrative reviews on the subject, seeking any other potentially eligible studies.

The articles included could be in any language, with no date limits, and all types of study design and must investigate elderly patients with PAD and/or who received physiotherapy. Review articles, studies in which the intervention was cut short or results were inconclusive, and protocols for future studies were all excluded.

The search process was conducted during April and May of 2020 and all searches were run in English. The Boolean operators “AND” and “OR” were used to construct the search statements, which were adapted to meet each database’s requirements. The MeSH (Medical Subject Headings) descriptors used were “peripheral arterial disease”, “peripheral artery disease”, “exercise tolerance”, “elderly”, “quality of life”, “HRQOL”, “health related quality of life”, “life quality”, “physical therapy specialty”, “physiotherapy specialty”, and “physiotherapy”.

The research question was defined according to the PICO strategy, in which “P” stands for patient or problem (elderly people with PAD); “I” for the intervention studied (physiotherapy); “C” for comparison or control (not analyzed in this study); and “O” for the outcome of interest (exercise tolerance and QoL). The questions used to guide the study were therefore defined as follows: “Does PAD have negative impacts on exercise tolerance and QoL in the elderly? How can physiotherapy help to improve these changes?”

The Oxford Center for Evidence-based Medicine scale was used for processing and analyzing the data, assessing evidence level and recommendation grade per study type. Studies that were clinical trials were evaluated using the PEDro scale, which assesses randomized controlled studies in terms of their methodological quality, internal validity, and statistical description.

## RESULTS

**Figure 1:**
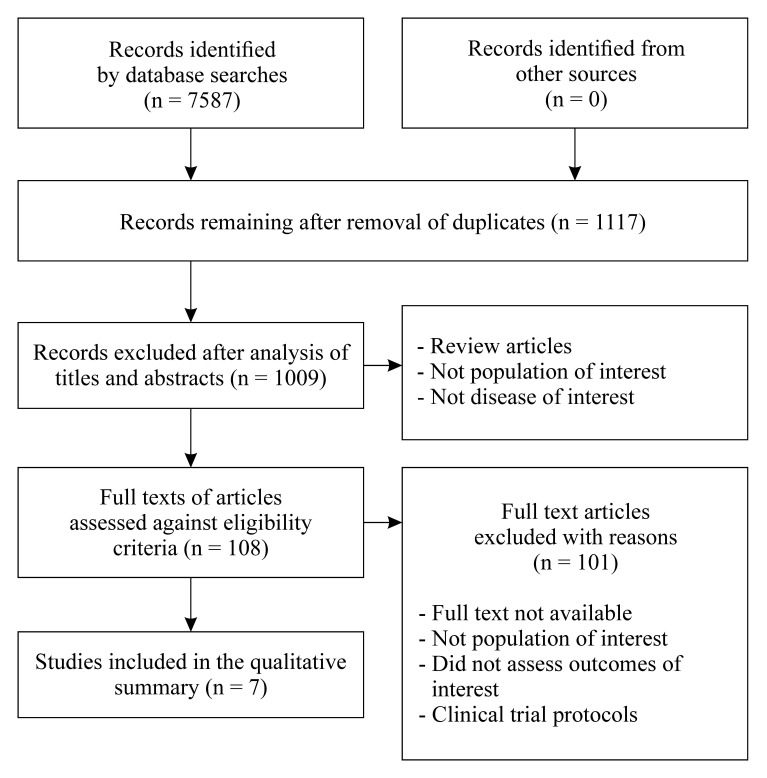
Flow diagram for the article search and selection process.

A total of 7,587 studies were identified on the following databases: MEDLINE (584), LILACS (1,873), SciELO (1,116), Scopus (1,603), PEDro (44), and Science Direct (2,367). After applying the eligibility criteria, seven articles were selected for the review. A flowchart illustrating the search process is shown in Figure 1.

The characteristics of the studies selected were as follows: 71.44% were controlled clinical trials,^9^^-^ 13 14.3% were uncontrolled clinical trials,^14^ and 14.3% were cross-sectional case-control studies.^15^ A total of 685 patients participated in the studies analyzed, a majority of whom were male (77.7%). Table 1 contains a summary of the main findings.

**Table 1:** Characteristics of the studies selected.

**Authors, country**	**Type of study**	**Population**	**Assessments**	**Interventions**
Langbein et al.,^9^ United States	Controlled RCT	n = 52 (51 M and 1 W), mean age: 67.05±7.95 years	Exercise tolerance: incremental load treadmill test, constant load treadmill test, and VO_2_max analysis	G1: Nordic walking 3x per week for 24 weeks.
				G2: control group, no exercise.
Tew et al.,^10^ United Kingdom	Controlled RCT	n = 57 (57 M and 0 W), mean age: 70±8 years	Exercise tolerance: test on cycle ergometer with electronic break, hand crank, VO_2_max analysis_,_ blood lactate concentration, and incremental treadmill test	G1: intermittent aerobic exercise (2 min exercise and 2 min rest) on hand crank ergometer, 2x per week, from 20 to 40 min per day, for 12 weeks.
				G2: control group, no exercises.
Malagoni et al.,^14^ Italy	Uncontrolled RCT	n = 289 (210 M and 79 W), mean age: 71±10.1 years	Exercise tolerance: constant load treadmill test.	All participants: 10 min walk 2x per day, 6 days per week, for 2 years. Afterwards, analysis of variables compared participants with good compliance, 13±2months (G1) with those with poor compliance, 12±2 months (G2).
			Quality of life: with SF-36 questionnaire.	
Collins et al.,^11^ United States	Controlled RCT	n = 103 (89 M and 14 W), mean age: 69.7±8.9 years	Exercise tolerance: incremental load treadmill test, constant load treadmill test (before and after 6, 12, and 24 weeks) and WIQ. Tissue oxygenation: with NIRS.	Interval exercises (low and high intensity) 3x per week for 24 weeks.
				G1: Nordic walking.
				G2: traditional walking.
			Quality of life: with SF-36 questionnaire.	
Dziubek et al.,^15^ Poland	Cross-sectional case-control study	n = 135 (83 M and 52 W), G1: 85 seniors with PAD and G2: 50 seniors without PAD, mean age: 70.35±7.7 years	Exercise tolerance: 6WT, plus assessment of lower limb dynamic muscle strength and peak torque with an isokinetic dynamometer	NA
Lamberti et al.,^12^ Italy	Controlled RCT	n = 27 (21 M and 6 W), mean age: 67±7 years	Exercise tolerance and claudication: constant load treadmill test and 6WT.	G1: home exercise, intermittent walking (1 min of exercise and 1 min of rest) 10 min per day, 6x per week, for 16 weeks.
			Quality of life: with SF-36 questionnaire. Also analyzed the cost/effectiveness of treatment and compliance.	G2: open surgery or revascularization or both, plus instructed to remain active.
Akerman et al.,^13^ New Zealand	Controlled RCT	n = 22 (15 H and 7 W), mean of 75.3±8.9 years	Exercise tolerance: walking test, 6WT.	G1: heat therapy with immersions in a heated swimming pool (~39 °C) from 3 to 5x per week, plus resistance exercises and calisthenics wearing warm clothes, for 15 to 30 min.
			Internal temperature during physical activity: telemetry capsule.	
			Oxygenation and volume of blood, vascular function: with FMD, NIRS, PWV, venous occlusion plethysmography, and CO re-breathing.	G2: walking for 30 to 60 min, 2x per week.
				Both groups exercised for 12 weeks.
			Quality of life: with SF-36 questionnaire.	
			Also analyzed compliance with program.	

CO = carbon monoxide; RCT = randomized clinical trial; FMD = flow-mediated dilation; G1 = group one; G2 = group two; M = men; W = women; min = minutes; n = number of participants; NA = not applicable; NIRS = near-infrared spectroscopy; SF-36 = Medical Outcomes Short-Form Health Survey; 6WT = 6-minute walk test; VO_2_max = maximum oxygen volume; PWV = pulse wave velocity; WIQ = Walking Impairment Questionnaire.

With regard to methodological quality, 85.72% of the articles met the requirements for assessment according to the PEDro criteria, with the exception being a single study,^15^ which had a cross-sectional case-control design. After evaluation, it was found that 71.44% of the studies had moderate scores for methodological quality and 28.56% had low scores, considering a maximum score of 10. The studies’ recommendation grades were high, indicating that they had an elevated level of recommendation confidence. The evidence levels, recommendation grades, and methodological quality scores for each study are shown in Table 2.

**Table 2:** Evidence level, recommendation grade, and methodological quality of the studies selected.

**Authors**	**Evidence level –recommendation grade**	**Methodological quality**
Langbein et al.^9^	1b – A	6/10
Tew et al.^10^	1c – A	3/10
Malagoni et al.^14^	1b – A	5/10
Collins et al.^11^	1b – A	5/10
Dziubek et al.^15^	1b – A	5/10
Lamberti et al.^12^	3b – B	NA
Akerman et al.^13^	1b – A	6/10

NA = not applicable.

## DISCUSSION

Analysis of the treatment protocols revealed a diverse range of approaches. Some studies, such as those by Langbein et al.^9^ and Collins et al.,^11^ adopted Nordic-style walking with the objective of increasing exercise tolerance, although they differed on the regularity and duration of their interventions. This type of walking is performed with the support of two poles, reducing impact on the lower limbs and also enabling more intense movement of the upper limbs and trunk.

In studies by Lamberti et al.^12^ and Malagoni et al.,^14^ used intermittent traditional walking sessions. The control group used for comparisons by Lamberti et al.^12^ comprised patients who underwent revascularization surgery. Finally, Tew et al.^10^ and Akerman et al.^13^ completely diverge from the others, using interventions consisting of upper limb aerobic exercises, immersion in hot water, resistance exercises, and calisthenics.

There were similarities in the assessment tools used. In general, participants were analyzed using the Medical Outcomes Short-Form Health Survey (SF-36) to measure QoL, in at least 57.14% of the studies. The outcome exercise tolerance was measured in all studies. The following tests and instruments were used for this: the Walking Impairment Questionnaire, incremental and constant load treadmill tests, upper limb ergometer testing, the 6WT, and maximum oxygen consumption (VO_2_max) analysis. 

The studies adopted the following methods to measure tissue oxygen perfusion and increases or reductions in body temperature provoked by the interventions: near-infrared spectroscopy, flow-mediated dilation, pulse wave velocity, venous occlusion plethysmography, carbon monoxide re-breathing, and telemetry capsules.

### Impact of PAD on exercise tolerance in the elderly and the role of physiotherapy

Dziubek et al.^15^ (n = 135) evaluated exercise tolerance and lower limb muscle strength in healthy elderly people and in elderly PAD patients. They concluded that PAD reduces exercise tolerance and functional capacity, significantly reducing strength and speed of muscle contraction (p < 0.005), distance covered before absolute claudication (p < 0.0001), distance covered in meters (p < 0.01), and gait velocity (p < 0.01).

Collins et al.^11^ enrolled elderly people with PAD who had an ankle-brachial index ≤ 0.90 or evidence of calcified vessels. The participants were divided into two groups: Nordic or traditional walking. The authors reported that both groups exhibited gradual improvement in tissue oxygenation over the course of the 24 weeks, but there were no differences in physical function or distance walked.

In contrast, Langbein et al.^9^ showed that Nordic walking significantly (p < 0.001) improved exercise tolerance, as attested by constant and incremental load treadmill tests, and also improved total distance walked (p < 0.001), gait velocity (p < 0.02), and VO_2_max (p = 0.01). Perceived pain levels had also reduced soon after participation in the training program.

Also using walking as the exercise method, Langbein et al.^9^ and Malagoni et al.^14^ assessed the effects of traditional walking. Both reported significant increase in distance walked before initial and absolute claudication and both gait velocity and pain threshold also improved. Malagoni et al.^14^ also reported that results were more significant in a group that had better compliance with the treatment.

Different types of intervention were adopted by two other studies. Tew et al.^10^ employed aerobic training on an arm-crank ergometer and, curiously, reported satisfactory walking performance results. The finding was attributed, at least in part, to the increased oxygen supply to the lower limbs. Notwithstanding, there were improvements in VO_2_max kinetics (from 44.7±10.4 to 41.3±14.4 seconds) and the minimum time to oxygen tissue saturation (from 268±305 to 410±366 seconds), which also increased significantly (p < 0.05) during treadmill exercise.

Akerman et al.^13^ used immersions in hot water (at approximately 39 °C), resistance exercises, and calisthenics, which led to increases in the distance covered before absolute claudication, from 350 m to 391 m (p = 0.006), and initial claudication, from 170 m to 213 m (p < 0.001). No significant changes were observed in blood volume, ankle-brachial index, functional results, or blood pressure; but, according to the authors, heat therapy was very well tolerated.

With regard to the contribution made by physiotherapy to the treatments employed, only Akerman et al.^13^ explicitly mentioned the role of a physiotherapist. The other studies did not specify the professional responsible for conducting the interventions.

### The impact of PAD on the QoL of elderly people and the role of physiotherapy

Just four studies^11^^-^ 14 (57.14%) analyzed the effectiveness of the interventions in terms of the elderly people’s QoL. The protocols proposed had varying effects on this outcome. The studies published by Lamberti et al.^12^ and Malagoni et al.^14^ both reported that the elderly people’s QoL improved in the physical and emotional domains.

In contrast, neither Collins et al.^11^ or Akerman et al.^13^ observed significant differences between groups. One probable reason that could explain these results is related to the fact that improved QoL in these patients was not necessarily dependent on the intervention proposed, but on performing physical exercises or undergoing surgical intervention, which corroborates what is postulated in the literature.

With regard to the emotional domain, only the study conducted by Akerman et al.^13^ reported a reduction in the score, of 35 points, irrespective of intervention. However, in this study, which used heat therapy by immersion in conjunction with resistance exercise and calisthenics, it was observed that scores for the pain domain were significantly lower (p = 0.041). No other significant changes in QoL were observed in the other domains of the SF-36.

Collins et al.^11^ used Nordic walking as treatment method, observing significant differences between the intervention and control groups in the physical and mental dimensions (p = 0.96 for the physical dimension and p = 0.43 for the mental dimension). The authors did not provide more detailed information on the SF-36 domains, such as the exact scores, for example.

Among the studies that employed a traditional walking intervention, Lamberti et al.^12^ highlighted that the treatment compliance rate was high (75%) and was reflected in the SF-36 scores, which improved significantly after conclusion of the rehabilitation program and were lower in a group of participants with worse compliance (25%).

These authors also reported that there was no significant difference between the groups in the questionnaire’s physical dimension and only the functional capacity domain revealed a significant increase in the group of participants who were treated with revascularization (p = 0.041), showing that the interventional treatment was more effective.

In a similar manner, Malagoni et al.^14^ reported a positive impact on patient QoL in all of the SF-36 domains, but particularly in the elements functional capacity (increased by 18.9 points) and pain (increased by 22.5 points). They observed that all of the SF-36 domain scores improved significantly after conclusion of the rehabilitation program, with statistically significant differences (p < 0.0001).

## CONCLUSIONS

The therapeutic interventions assessed in these studies contributed to improved exercise tolerance among the elderly participants, with increases in distance covered, gait velocity, and distance walked free from symptoms, regardless of the type of intervention employed. Moreover, some of the studies reported improvements in maximum functional capacity, measured using VO_2_max. These functional gains were reflected in positive changes to QoL, with higher total and domain scores on the SF-36 questionnaire.

It is important to point out that the protocols employed were not standardized, preventing identification of the superiority of any of the interventions over the others. Nevertheless, the majority of treatments included aerobic exercises. In summary, it is clear that the treatments described can benefit elderly people with PAD, but there is a need to standardize intervention protocols, in order to facilitate compilation of guidelines to help in the clinical practice of professionals who work with these patients.
