# Supplementary material for: The impact of peripheral arterial disease on exercise tolerance and quality of life in the elderly and the role of cardiovascular physiotherapy: review article
Source: J Vasc Bras. 2021 Apr 28;20:e20200117. doi: 10.1590/1677-5449.200117 (PMC8147882; doi:10.1590/1677-5449.200117)
Supplement: Translation

# Translation: Repercussão da doença arterial periférica na tolerância ao exercício e na qualidade de vida de idosos e o papel da fisioterapia cardiovascular: artigo de revisão

**Authors:** Ana Leticia Gonçalves Lourenço, Josicléia Leôncio da Silva, Jéssica Costa Leite

**Affiliations:** 1 Centro Universitário UNIFACISA, Departamento de Fisioterapia, Campina Grande, PB, Brasil.

**Keywords:** doença arterial periférica, tolerância ao exercício, qualidade de vida

## Abstract

A doença arterial periférica tem como principal sintoma a claudicação intermitente, fator que resulta em incapacidade funcional, comprometendo a qualidade de vida. Esta revisão objetivou fazer um levantamento sobre os impactos ocasionado pela doença nos idosos, investigando as possíveis contribuições da fisioterapia cardiovascular. Para isso, realizou-se uma busca nas bases de dados MEDLINE, LILACS, SciELO, Scopus, Science Direct e PEDro, a qual identificou 7.587 estudos. Desses, sete atenderam aos critérios de elegibilidade, sendo então agrupados e analisados conforme o nível de evidência, grau de recomendação e qualidade metodológica. Pôde-se observar um considerável impacto da doença sobre a tolerância ao exercício e a qualidade de vida. Quanto à abordagem terapêutica desses desfechos, os estudos relataram que houve melhora na deambulação e na qualidade de vida, aumento da capacidade funcional e redução da dor. No que se refere à modalidade de tratamento, a maioria das pesquisas incluíram exercícios aeróbicos.

## INTRODUÇÃO

A doença arterial obstrutiva periférica pode causar obstrução parcial ou total das artérias dos membros inferiores, provocando redução do fluxo sanguíneo para as extremidades. Nos casos mais críticos, existe o risco de amputação do membro, caso não haja possibilidade de revascularização^1^^,^ 2.

Aproximadamente 20% da população acometida pela doença têm mais de 65 anos de idade. No Brasil, estima-se que anualmente 0,053% da população seja diagnosticada com doença arterial periférica (DAP), incluindo homens com idade entre 55 e 74 anos e mulheres de 65 a 74 anos. Essa doença pode progredir de forma assintomática em até 80% dos casos, o que atrasa e dificulta o diagnóstico precoce, comprometendo o prognóstico^3^^,^ 4.

Dentre os principais fatores de risco para DAP, destacam-se: diabetes, tabagismo, hipertensão e dislipidemia, condições preponderantes na população idosa^5^. Quanto à apresentação clínica, destaca-se a claudicação intermitente, a qual acomete cerca de um terço dos pacientes. Além disso, são frequentes sintomas como cãibra, dor ou cansaço nos membros inferiores (MMII), que pioram no decorrer da caminhada e aliviam no repouso^6^.

Em estágios mais avançados da DAP, pode ocorrer necrose tecidual, aumentando significativamente o risco de o indivíduo perder o membro acometido^6^. Todos esses fatores fazem com que a DAP seja responsável pela redução da capacidade funcional e pela piora da qualidade de vida (QV) dos pacientes^1^^,^ 3.

Dessa forma, é recomendada a avaliação vascular precoce nesses indivíduos, a fim de que se determine a capacidade e a tolerância ao exercício. Para isso, podem ser utilizadas medidas diretas, por meio do teste de esteira ou do teste de caminhada de seis minutos (TC6), assim como medidas indiretas, utilizando escalas específicas^7^. Já a QV pode ser avaliada por questionários que consideram tanto aspectos físicos, psicológicos e sociais como a percepção do indivíduo^8^.

Nesse contexto, a fisioterapia apresenta um importante papel no tratamento da DAP, pois pode atuar no pré-operatório com o objetivo de controlar a dor, reduzir edemas, aumentar a amplitude de movimento, estimular a deambulação e propiciar a educação em saúde; e no pós-operatório com o objetivo de promover ganho de força muscular, amplitude de movimento e capacidade funcional, por meio de exercícios resistidos, aeróbicos e de flexibilidade^2^.

Diante do exposto, considerando o crescente número de idosos acometidos pela DAP no Brasil, pesquisar sobre essa temática, em busca de informação acerca das terapêuticas existentes e das alterações relacionadas à doença, pode elevar as chances de sucesso no tratamento. Portanto, este trabalho se propôs a revisar a literatura a fim de verificar o impacto da DAP na tolerância ao exercício e na QV de idosos, além de investigar as possíveis contribuições da fisioterapia para minimizar essas alterações.

## MÉTODOS

Trata-se de uma revisão integrativa da literatura, em que se realizou uma busca por artigos indexados nas bases de dados MEDLINE/Pubmed, LILACS, SciELO, Scopus (Elsevier), Science Direct e PEDro. Foi realizada também a busca manual nas referências dos artigos incluídos nesta pesquisa e das revisões integrativas já existentes sobre essa temática, em busca de quaisquer outros estudos potencialmente elegíveis.

Incluíram-se na pesquisa artigos em qualquer idioma, sem restrição de ano, com todos os tipos de delineamento, que avaliaram pacientes idosos com DAP e/ou que realizaram atendimento fisioterapêutico. Excluíram-se artigos de revisão, estudos em que a intervenção tinha sido interrompida ou com resultados inconclusivos, e protocolos de estudos futuros.

O levantamento foi realizado em abril e maio de 2020, e todas as buscas foram executadas em inglês. Utilizaram-se os operadores booleanos “AND” e “OR” para montar as estratégias de busca, que foram adaptadas conforme as exigências de cada base. Os descritores MeSH (Medical Subject Headings) adotados foram: “peripheral arterial disease”, “peripheral artery disease”, “exercise tolerance”, “elderly”, “quality of life”, “HRQOL”, “health related quality of life”, “life quality”, “physical therapy specialty”, “physiotherapy specialty” e “physiotherapy”.

A questão de pesquisa foi fundamentada por meio da estratégia PICO, na qual “P” refere-se ao paciente ou problema (idosos com DAP); “I” à intervenção estudada (fisioterapia); “C” à comparação ou controle (não foi considerado neste estudo); e “O” ao desfecho de interesse (tolerância ao exercício e QV). Portanto, a pergunta norteadora para a condução da presente pesquisa foi: “A DAP traz repercussões negativas na tolerância ao exercício e na QV dos idosos? De que forma a fisioterapia pode auxiliar na melhora dessas alterações?”.

Para o processamento e análise dos dados, foi utilizada a escala *Oxford Centre for Evidence-based Medicine*, a fim de avaliar o nível de evidência e o grau de recomendação por tipo de pesquisa. Já para avaliar os estudos selecionados que se caracterizaram como ensaios clínicos, foi utilizada a escala PEDro, a qual avalia estudos controlados aleatorizados em termos de qualidade metodológica, validade interna e descrição estatística.

## RESULTADOS

Foram identificados 7.587 estudos, obtidos nos seguintes bancos de dados: MEDLINE (584), LILACS (1.873), SciELO (1.116), Scopus (1.603), PEDro (44) e Science Direct (2.367). Após a aplicação dos critérios de elegibilidade, sete artigos foram selecionados para compor esta pesquisa. O esquema dos resultados das buscas está representado na Figura 1.

**Figura 1:** Fluxograma do processo de busca e seleção dos artigos.

Quanto às características dos estudos selecionados, 71,44% eram ensaios clínicos controlados^9^^-^ 13, 14,3% eram ensaios clínicos não controlados^14^, e 14,3% eram estudos transversais do tipo caso-controle^15^. Ao todo, 685 pacientes fizeram parte dos estudos analisados, em sua maioria indivíduos do sexo masculino (77,7%). Uma síntese dos principais achados está representada na Tabela 1.

**Tabela 1:** Caracterização dos estudos selecionados.

**Autor, país**	**Tipo de estudo**	**População**	**Avaliação**	**Intervenção**
Langbein et al.^9^, EUA	ECR controlado	n = 52 (51 H e 1 M), média de 67,05±7,95 anos	Tolerância ao exercício: teste de esteira com carga incremental, carga constante e análise do VO_2_max	G1: caminhada nórdica 3x por semana durante 24 semanas.
				G2: grupo controle, sem exercícios.
Tew et al.^10^, Reino Unido	ECR controlado	n = 57 (57 H e 0 M), média de 70±8 anos	Tolerância ao exercício: teste em cicloergômetro com freio eletrônico, manivela de braço, análise do VO_2_max_,_ concentração de lactato no sangue e teste incremental em esteira	G1: exercício aeróbico intermitente (2 min de exercício e 2 de repouso) em ergômetro de MMSS, 2x por semana, de 20 a 40 min por dia, durante 12 semanas.
				G2: grupo controle, sem exercícios.
Malagoni et al.^14^, Itália	ECR não controlado	n = 289 (210 H e 79 M), média de 71±10,1 anos	Tolerância ao exercício: teste de esteira com carga constante.	Todos os participantes: caminhada de 10 min 2x por dia, 6 dias por semana, durante 2 anos. Posteriormente, a análise das variáveis foi realizada entre os participantes que tiveram alta adesão, 13±2meses (G1) com os de baixa adesão, 12±2 meses (G2).
			Qualidade de vida: com questionário SF-36.	
Collins et al.^11^, EUA	ECR controlado	n = 103 (89 H e 14 M), média de 69,7±8,9 anos	Tolerância ao exercício: teste de esteira com carga incremental, carga constante (antes e após 6, 12 e 24 semanas) e WIQ. Oxigenação tecidual: com NIRS.	Exercício intervalado (baixa e alta intensidade) 3x por semana durante 24 semanas.
				G1: caminhada nórdica.
				G2: caminhada tradicional.
			Qualidade de vida: com questionário SF-36.	
Dziubek et al.^15^, Polônia	Estudo transversal do tipo caso-controle	n = 135 (83 H e 52 M), G1: 85 idosos com DAP e G2: 50 idosos sem DAP, média de 70,35±7,7 anos	Tolerância ao exercício: TC6, mais avaliação da força muscular dinâmica dos MMII e pico de torque com dinamômetro isocinético	NA
Lamberti et al.^12^, Itália	ECR controlado	n = 27 (21 H e 6 M), média de 67±7 anos	Tolerância ao exercício e claudicação: teste de esteira com carga constante e TC6.	G1: exercício domiciliar, caminhada intermitente (1 min de exercício e 1 de repouso) 10 min ao dia, 6x por semana, durante 16 semanas.
			Qualidade de vida: com questionário SF-36. Também avaliou o custo/efetividade do tratamento e adesão.	G2: cirurgia aberta ou revascularização ou ambas, mais orientações para permanecerem ativos.
Akerman et al.^13^, Nova Zelândia	ECR controlado	n = 22 (15 H e 7 M), média de 75,3±8,9 anos	Tolerância ao exercício: teste de caminhada, TC6.	G1: termoterapia com imersões em piscina aquecida (~39 °C) de 3 a 5x por semana, mais exercícios de resistência e calistênicos com roupas aquecidas, durante 15 a 30 min.
			Temperatura interna durante atividade física: cápsula de telemetria.	
			Oxigenação e volume do sangue, função vascular: com a FMD, NIRS, VOP, pletismografia de oclusão venosa e reinalação de CO.	G2: caminhade 30 a 60 min, 2x por semana.
				Ambos os grupos exercitaram-se durante 12 semanas.
			Qualidade de vida: com questionário SF-36.	
			Também avaliou a aderência ao programa.	

CO = monóxido de carbono; ECR = ensaio clínico randomizado; FMD = dilatação mediada por fluxo; G1 = grupo um; G2 = grupo dois; H = homens; M = mulheres; min = minutos; MMII = membros inferiores; MMSS = membros superiores; n = número de participantes; NA = não se aplica; NIRS = *near-infrared spectroscopy* (espectroscopia no infravermelho próximo); SF-36 = *Medical Outcomes Short-Form Health Survey*; TC6 = teste de caminhada de 6 minutos; VO_2_max = volume máximo de oxigênio; VOP = velocidade de onda de pulso; WIQ = *Walking Impairment Questionnaire*.

No que se diz respeito à qualidade metodológica, 85,72% dos artigos preencheram os requisitos para avaliação, conforme os critérios da escala PEDro, à exceção de um estudo^15^, por se tratar de uma pesquisa de caráter transversal do tipo caso-controle. Após a avaliação, evidenciou-se que 71,44% das pesquisas exibiram escores medianos de qualidade metodológica, e 28,56% exibiram escore baixo, considerando que a pontuação máxima é 10. O grau de recomendação dos artigos foi alto, indicando que os estudos selecionados apresentaram um elevado grau de confiança e recomendação. Os níveis de evidência, grau de recomendação e qualidade metodológica de cada estudo estão expostos na Tabela 2.

**Tabela 2:** Nível de evidência, grau de recomendação e qualidade metodológica dos estudos selecionados.

**Autor**	**Nível de evidência – grau de recomendação**	**Qualidade metodológica**
Langbein et al.^9^	1b – A	6/10
Tew et al.^10^	1c – A	3/10
Malagoni et al.^14^	1b – A	5/10
Collins et al.^11^	1b – A	5/10
Dziubek et al.^15^	1b – A	5/10
Lamberti et al.^12^	3b – B	NA
Akerman et al.^13^	1b – A	6/10

NA = não se aplica.

## DISCUSSÃO

Ao analisar os protocolos de tratamento, evidenciou-se uma diversidade nas abordagens utilizadas. Alguns estudos, como os de Langbein et al.^9^ e Collins et al.^11^, adotaram caminhadas nórdicas visando potencializar a tolerância ao exercício, embora divergindo quanto à periodicidade e à duração da intervenção. Essa modalidade de caminhada é realizada apoiando-se em dois bastões, o que reduz o impacto nos MMII ao mesmo tempo em que permite a movimentação mais intensa dos membros superiores (MMSS) e do tronco.

Já as pesquisas de Lamberti et al.^12^ e Malagoni et al.^14^ utilizaram sessões intermitentes de caminhadas tradicionais. O grupo controle formado por Lamberti et al.^12^ era composto por pacientes submetidos a cirurgia de revascularização. Por fim, Tew et al.^10^ e Akerman et al.^13^ divergiram totalmente dos anteriores, pois a intervenção usada consistia em exercícios aeróbicos de MMSS, imersões em água quente, exercícios resistidos e calistênicos.

Quanto às ferramentas de avaliação, houve semelhanças. Em geral, os indivíduos foram analisados pelo questionário *Medical Outcomes Short-Form Health Survey* (SF-36), a fim de mensurar a QV, em pelo menos 57,14% dos estudos. Já o desfecho tolerância ao exercício foi mensurado em todas as pesquisas. Para isso, foram utilizados os seguintes instrumentos: *Walking Impairment Questionnaire*, testes de esteira com carga incremental e constante, teste em ergômetro de MMSS, TC6 e análise do consumo máximo de oxigênio (VO_2_max).

No que se refere à avaliação da perfusão de oxigênio nos tecidos e do aumento ou diminuição de temperatura corporal promovidos pela intervenção proposta, os estudos adotaram os seguintes métodos de mensuração: espectroscopia no infravermelho próximo, dilatação mediada por fluxo, velocidade de onda de pulso, pletismografia de oclusão venosa, reinalação de monóxido de carbono e cápsula de telemetria.

### Impacto da DAP na tolerância ao exercício de idosos e o papel da fisioterapia

O estudo de Dziubek et al.^15^ (n = 135) avaliou a tolerância ao exercício e a força dos músculos dos MMII em idosos saudáveis e com DAP. Os autores concluíram que a DAP reduz a tolerância ao exercício e a capacidade funcional, devido à diminuição significativa da força e da velocidade de contração muscular (p < 0,005), da distância para claudicação absoluta (p < 0,0001), da distância percorrida em metros (p < 0,01) e da velocidade da marcha (p < 0,01).

Já a pesquisa de Collins et al.^11^ incluiu idosos com DAP que apresentavam índice tornozelo-braquial ≤ 0,90 ou alguma evidência de vasos calcificados. Os participantes foram divididos em dois grupos: caminhada nórdica ou tradicional. Os autores relataram que ambos os grupos apresentaram melhora gradativa na oxigenação tecidual durante 24 semanas, porém não houve diferenças na função física ou na distância percorrida.

Entretanto, Langbein et al.^9^ mostraram que a caminhada nórdica melhorou significativamente (p < 0,001) a tolerância ao exercício, verificada pelos testes de carga constante e incremental em esteira, e também a distância total percorrida (p < 0,001), a velocidade da caminhada (p < 0,02) e o VO_2_max (p = 0,01). Houve também redução dos níveis de percepção de dor logo após a aplicação do programa de treinamento.

Ainda utilizando o exercício de caminhada, Langbein et al.^9^ e Malagoni et al.^14^ avaliaram seus efeitos, porém na modalidade tradicional. Ambos retrataram aumento significativo na distância para claudicação inicial e absoluta, com acréscimo também na velocidade da marcha e no limiar da dor. Malagoni et al.^14^ ainda destacaram que os resultados foram mais significativos no grupo que melhor aderiu ao programa de tratamento.

Uma forma diferente de intervenção foi adotada por outras duas pesquisas. Tew et al.^10^ utilizaram o treino aeróbico de MMSS realizado em ergômetro e, curiosamente, relataram resultados satisfatórios no desempenho da caminhada. Esse achado foi atribuído, pelo menos em parte, ao aumento da oferta de oxigênio aos MMII. Ainda assim, houve melhora da cinética do VO_2_max (de 44,7±10,4 para 41,3±14,4 segundos) e do tempo mínimo para saturação de oxigênio tecidual (de 268±305 para 410±366 segundos), que também aumentou significativamente (p < 0,05) durante a caminha em esteira.

Já Akerman et al.^13^ utilizaram imersões (a aproximadamente 39 °C), exercícios resistidos e calistênicos, que resultaram no aumento da distância para claudicação absoluta de 350 m para 391 m (p = 0,006) e da claudicação inicial, que passou de 170 m para 213 m (p < 0,001). Não foram observadas alterações significativas em termos de volume sanguíneo, índice tornozelo-braquial, resultados funcionais ou níveis pressóricos; porém, a adesão e a tolerância à termoterapia foram excelentes, segundo os autores relataram.

No que se refere às contribuições da fisioterapia para o tratamento, apenas o estudo de Akerman et al.^13^ mencionou claramente o papel do fisioterapeuta. Os demais não relataram qual profissional ficou responsável por conduzir a intervenção.

### Impacto da DAP na QV de idosos e o papel da fisioterapia

Apenas quatro pesquisas^11^^-^ 14 (57,14%) abordaram a efetividade da intervenção na QV dos idosos. O efeito dos protocolos propostos sobre esse desfecho foi variável. Os estudos realizados por Lamberti et al.^12^ e Malagoni et al.^14^ relataram que houve melhora na QV dos idosos, no que se refere aos domínios das dimensões física e emocional.

Por outro lado, Collins et al.^11^ e Akerman et al.^13^ não observaram diferenças significantes entre os grupos. Uma provável explicação para os resultados obtidos pode estar relacionada ao fato de que a melhora na QV desses pacientes não esteve necessariamente atrelada à intervenção proposta, mas sim à execução dos exercícios físicos ou à intervenção cirúrgica, fato que corrobora o postulado pela literatura.

Quanto ao domínio emocional, apenas o estudo de Akerman et al.^13^ apresentou redução de 35 pontos no escore, independentemente da intervenção. Todavia, observou-se que o domínio dor foi significativamente menor (p = 0,041), sendo que a pesquisa utilizou termoterapia por imersão associada à prática de exercícios de resistência e calistênicos. Não foram observadas outras alterações significativas na QV para os demais domínios do SF-36.

Em relação às dimensões física e mental, Collins et al.^11^, que utilizaram como abordagem terapêutica a caminhada nórdica, não observaram diferenças significativas entre os grupos intervenção e controle (p = 0,96 para a dimensão física e p = 0,43 para a dimensão mental). Informações mais detalhadas sobre os domínios do SF-36, como os valores exatos dos escores, não foram relatadas pelos autores.

Entre as pesquisas que adotaram a caminhada tradicional, Lamberti et al.^12^ destacaram que a adesão ao tratamento foi alta (75%), repercutindo nos escores do SF-36, os quais melhoraram significativamente após a conclusão do programa de reabilitação, sendo menores no grupo de participantes com baixa adesão (25%).

Os autores ainda acrescentam que não houve diferença significativa entre os grupos na dimensão física do questionário, e que somente o domínio capacidade funcional revelou um aumento significativo no grupo de participantes que foram submetidos a revascularização (p = 0,041), mostrando que o tratamento intervencionista foi mais efetivo.

De maneira semelhante, Malagoni et al.^14^ mostraram um impacto positivo na QV dos pacientes em todos os domínios do SF-36, principalmente em relação às áreas de capacidade funcional (aumento de 18,9 pontos) e dor (aumento de 22,5 pontos). Pôde-se observar que todos os escores dos domínios do SF-36 melhoraram significativamente após a conclusão do programa de reabilitação, exibindo valores estatisticamente significativos (p < 0,0001).

## CONCLUSÕES

As intervenções terapêuticas avaliadas nos estudos contribuíram para melhora da tolerância ao exercício em idosos, com aumento da distância percorrida, da velocidade da marcha e da distância livre de sintomas, independente da modalidade empregada. Além disso, alguns estudos relataram que houve melhora na capacidade funcional máxima, medida por meio do VO_2_max. Esses ganhos funcionais refletiram positivamente na QV, com melhora nos escores totais e por domínio do questionário SF-36.

É importante ressaltar que não houve padronização dos protocolos utilizados, o que inviabiliza a identificação da superioridade de alguma intervenção em relação a outra. No entanto, a maioria dos tratamentos incluíram exercícios aeróbicos. Em suma, fica claro que as terapias apresentadas podem beneficiar idosos com DAP; porém, se faz necessária uma padronização dos protocolos de intervenção, de modo a favorecer a elaboração de diretrizes que auxiliem na prática clínica dos profissionais que atuam com esse perfil de pacientes.
